# Liver impairment and medical management of Cushing syndrome and MACS

**DOI:** 10.3389/fendo.2025.1660316

**Published:** 2025-10-24

**Authors:** Mari Minasyan, Wiktoria Suchy, Andrzej Fedak, Aleksandra Gamrat-Żmuda, Alicja Hubalewska-Dydejczyk, Elena Valassi, Aleksandra Gilis-Januszewska

**Affiliations:** ^1^ Chair and Department of Endocrinology, Jagiellonian University Medical College, Kraków, Poland; ^2^ Students’ Scientific Circle of Department of Endocrinology, Jagiellonian University Medical College, Kraków, Poland; ^3^ Department of Radiology, Jagiellonian University Medical College, Kraków, Poland; ^4^ Endocrinology Department, Hospital Germans Trias i Pujol, Badalona, Spain; ^5^ Universitat Internacional de Catalunya (UIC), Barcelona, Spain

**Keywords:** Cushing, MACS, liver steatosis, liver fibrosis, steroidogenesis inhibitors, osilodrostat, metyrapone, hypercortisolemia

## Abstract

Cushing syndrome (CS) and mild autonomous cortisol secretion syndrome (MACS) are states of endogenous hypercortisolemia, associated with multiple metabolic complications. The data on the impact of cortisol on the liver are inconsistent at times. From one perspective, some studies proved hepatotoxic cortisol action. Elevated liver enzymes and liver steatosis are common findings in patients with newly diagnosed CS and MACS (liver steatosis prevalence: 20%–66% and 25%–57%, respectively). Normocortisolemic subjects with liver steatosis/metabolic-associated steatohepatitis seem to have higher cortisol concentration than the healthy population. In contrast, other studies suggest that the liver impairment prevalence in hypercortisolemic patients with so many metabolic comorbidities would be expected to be much higher than it is reported. They postulate anti-inflammatory cortisol action as a preventive factor for liver disease progression in subjects with CS and MACS. The data on the hepatic safety profile of hypercortisolemia pharmacotherapy seems to be conflicting at times. Antihypercortisolemic medical therapy can potentially cause liver impairment; therefore, implementing the treatment of hypercortisolemia is often challenging in patients with liver dysfunction. We present two CS cases with baseline liver impairment, which improved on the treatment with steroidogenesis inhibitors. The case reports are followed by literature review regarding liver dysfunction in endogenous hypercortisolemia, impact of hypothalamic–pituitary–adrenal axis on the liver, and liver safety profile of medical treatment used in endogenous hypercortisolemia.

## 1 Introduction

Cushing syndrome (CS) and mild autonomous cortisol secretion syndrome (MACS) are conditions caused by cortisol excess. CS is a state of overt hypercortisolemia with marked physical symptoms. MACS is a state of milder degree of hypercortisolemia, without physical signs of cortisol excess. To different degrees, CS and MACS are associated with cardiovascular, thrombotic, metabolic, infectious, musculoskeletal, and psychiatric complications, which increase mortality rate and impair quality of life among these patients (1–5). The data on the impact of hypothalamic–pituitary–adrenal (HPA) axis on the liver function are divergent at times. Elevated liver enzymes are common baseline findings in patients with CS (4). CS and MACS are associated with an increased prevalence of liver steatosis (LS; 20%–66% and 25%–57%, respectively), classified as metabolic dysfunction-associated steatotic liver disease [MASLD, formerly known as nonalcoholic fatty liver disease (NAFLD)] (6–13). On the other hand, cortisol’s anti-inflammatory action may play a protective role, and the rate of LS in CS may increase after management of active hypercortisolemia (14).

Untreated LS may evolve to hepatitis [metabolic dysfunction-associated steatohepatitis (MASH), formerly classified as nonalcoholic steatohepatitis (NASH)] and fibrotic sequelae. To the best of our knowledge, the incidence of liver fibrosis (LF) in CS has been so far evaluated in two studies that used Fibroscan (3.4%) and blood-based scores (15.4%) (15, 16). Currently, there are two ongoing prospective studies investigating liver images on magnetic resonance imaging (MRI) scans in newly diagnosed patients with CS and 1 year after successful treatment (15, 16). One of them investigates LS by using liver MRI (15); another one measures LF using MRI elastography (MRE) (16).

The reports on the liver safety profile of some of the antihypercortisolemic medications seem to be inconsistent at times. Therefore, implementing the treatment of hypercortisolemia may sometimes seem challenging in patients with CS and MACS with hepatic dysfunction.

In this paper, we present two CS cases with significant liver impairment treated with steroidogenesis inhibitors followed by literature review regarding liver dysfunction in endogenous hypercortisolemia, impact of HPA axis on the liver, and liver safety profile of medical treatment used in endogenous hypercortisolemia.

## 2 Case reports

### 2.1 Case 1

**Figure 1:**
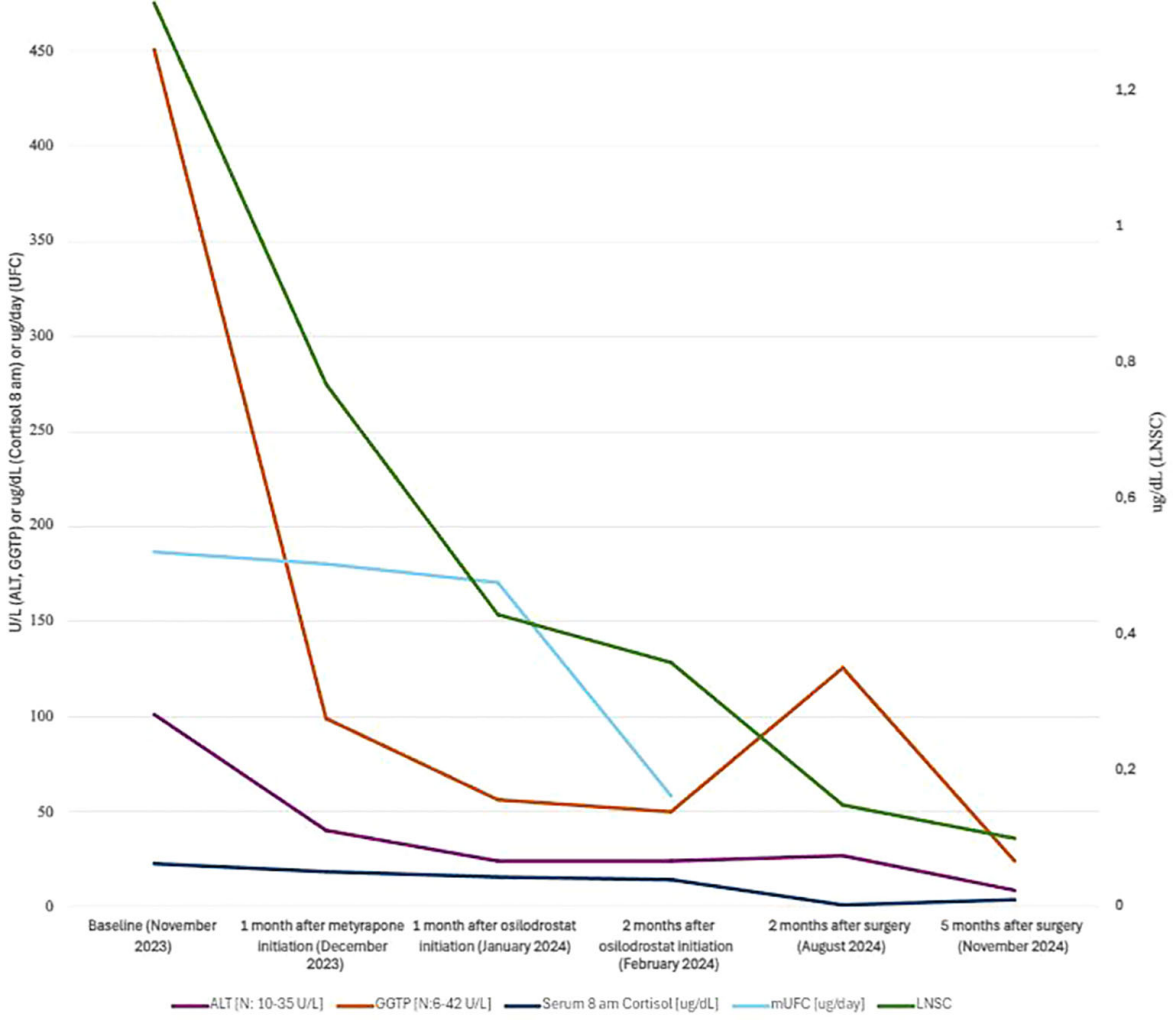
Case 1- Graph presenting LFT and cortisolemia level in the course of adrenostatic therapy.

A 51-year-old overweight woman with a 3-year history of diabetes mellitus (DM), hypertension, and LS was referred to the endocrinology department due to worsening glycemic and blood pressure control. On admission, the patient reported insomnia, hot flushes, and muscle weakness for 1 year. Physical examination revealed abdominal obesity, moon face, dorsocervical fat pad, abdominal striae, and skin bruises. Laboratory evaluation showed poorly controlled DM and hypercholesterolemia. Hepatic function evaluation indicated significantly elevated liver function tests [LFTs; up to 11× upper reference limit (URL)] without liver function decompensation (Child-Pugh A—the score is characterized in the **Supplementary File 1**). Insulinotherapy, escalation of hepatoprotective medications (ursodeoxycholic acid and ornithine aspartate), and antihypertensives were implemented. Hormonal evaluation confirmed adrenocorticotropic hormone (ACTH)-dependent CS. Metyrapone was started at the daily dose of 750 mg for 1 month, substituted by osilodrostat at the initial daily dose of 4 mg. After 1 month of metyrapone therapy, a significant decrease of hypercortisolemia (halving of serum midnight cortisol and late-night salivary cortisol) and an LFT drop (up to 2.7× URL) and amelioration of metabolic control were observed. The decision to replace metyrapone with osilodrostat was made for the patient’s convenience (easier dosing). Two months of osilodrostat treatment resulted in further improvement of cortisolemia, metabolic control, and almost complete LFT normalization. Inferior petrosal sinus sampling suggested right side pituitary microadenoma. After 7 months of treatment with steroidogenesis inhibitors, the patient underwent a successful transsphenoidal surgery of pituitary adenoma. LFT normalized 5 months after the surgical treatment of Cushing disease (CD). The results are presented in **Table 1** and **Figure 1**.

**Table 1:** Case 1—Test results evaluating cortisolemia level, liver enzymes, diabetes mellitus, and dyslipidemia control (baseline, during adrenostatic treatment, and after surgery).

	Baseline (Nov 2023)	1 Month of M therapy (Dec 2023)	1 Month of O therapy (Jan 2024)	2 Months of O therapy (Feb 2024)	2 Months after surgery (Aug 2024)	5 Months after surgery (Nov 2024)	7 Months after surgery (Jan 2025)
ALT [U/L]	101	40	24	24	27	9	9
AST [U/L]	39	21	16	18	27		12
GGT [U/L]	451	99	56	50	126	24	19
Bilirubine mmol/L	8.19	5.81	4.33	4.03	6.48		4.06
6AM COR [μg/dL]	22.2		18.3	13.9	0.41		
8AM COR [μg/dL]	23	18.2	15.7	14.2	0.78	3.96	3.11
24AM COR [μg/dL]	23.3	12	13.4	13.6	0.13		
LNSC [μg/dL]	1.33	0.77	0.43	0.36			
mUFC [μg/day]	186.3		170.45	58.4			
HBA1C [%]	14.4	11.1	9.3	8.4	9.5		6.2
TC mmol/L	7.1	5.9		4.8	5.4		4.8
LDL-C [mmol/L]	6.11	5.02		3.86	4.57		
Non-HDL-C [mmol/L]	4.82	4.03		3.41	2.6		3.84
TAG [mmol/L]	5.72	2.63		1.95	4.33		2.6
Weight [kg]	84	84	84	84			

Laboratory reference ranges: ALT [N: 10–35 U/L], AST [N: 10–35 U/L], GGT [N: 6–42 U/L], LNSC [<0.274 μg/dL], HBA1C [N <6.4%], non-HDL-C [N < 2.2 mmol/L], LDL-C [N < 1.8 mmol/L], TC [N < 5.2 mmol/L], mUFC [N: 10–100 μg/day], TAG [N < 2.30 mmol/L].

### 2.2 Case 2

**Figure 2:**
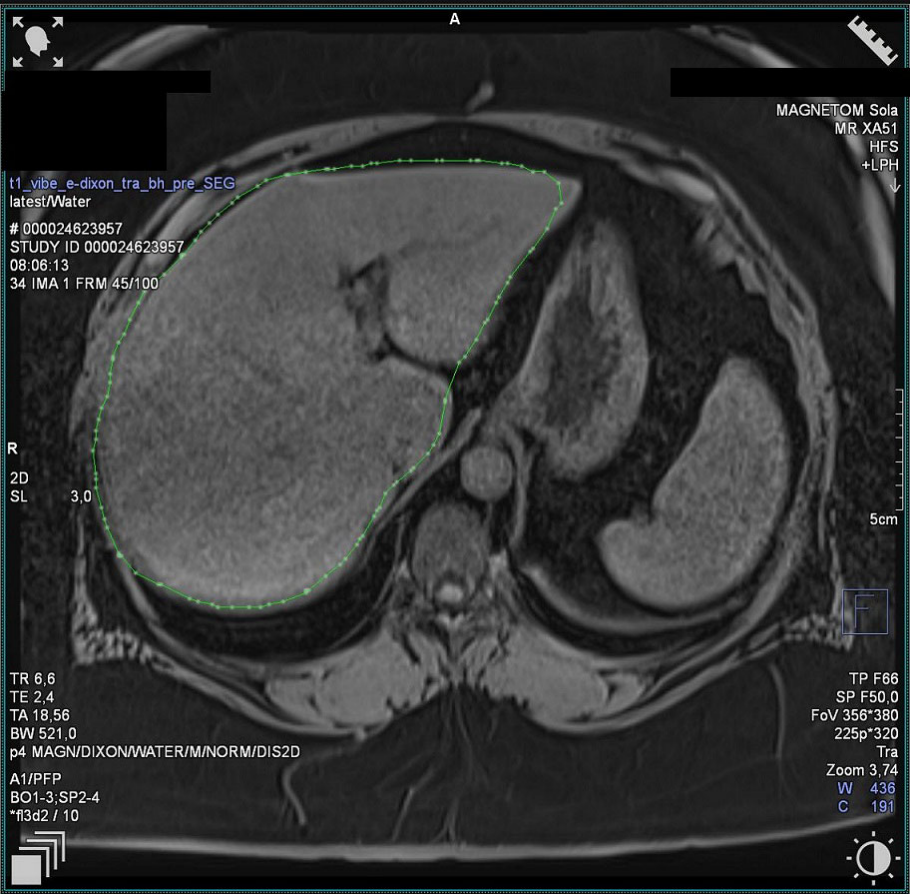
Case 2- Liver MRI image 1 (19.3% of fat content, N<5%).

**Figure 3:**
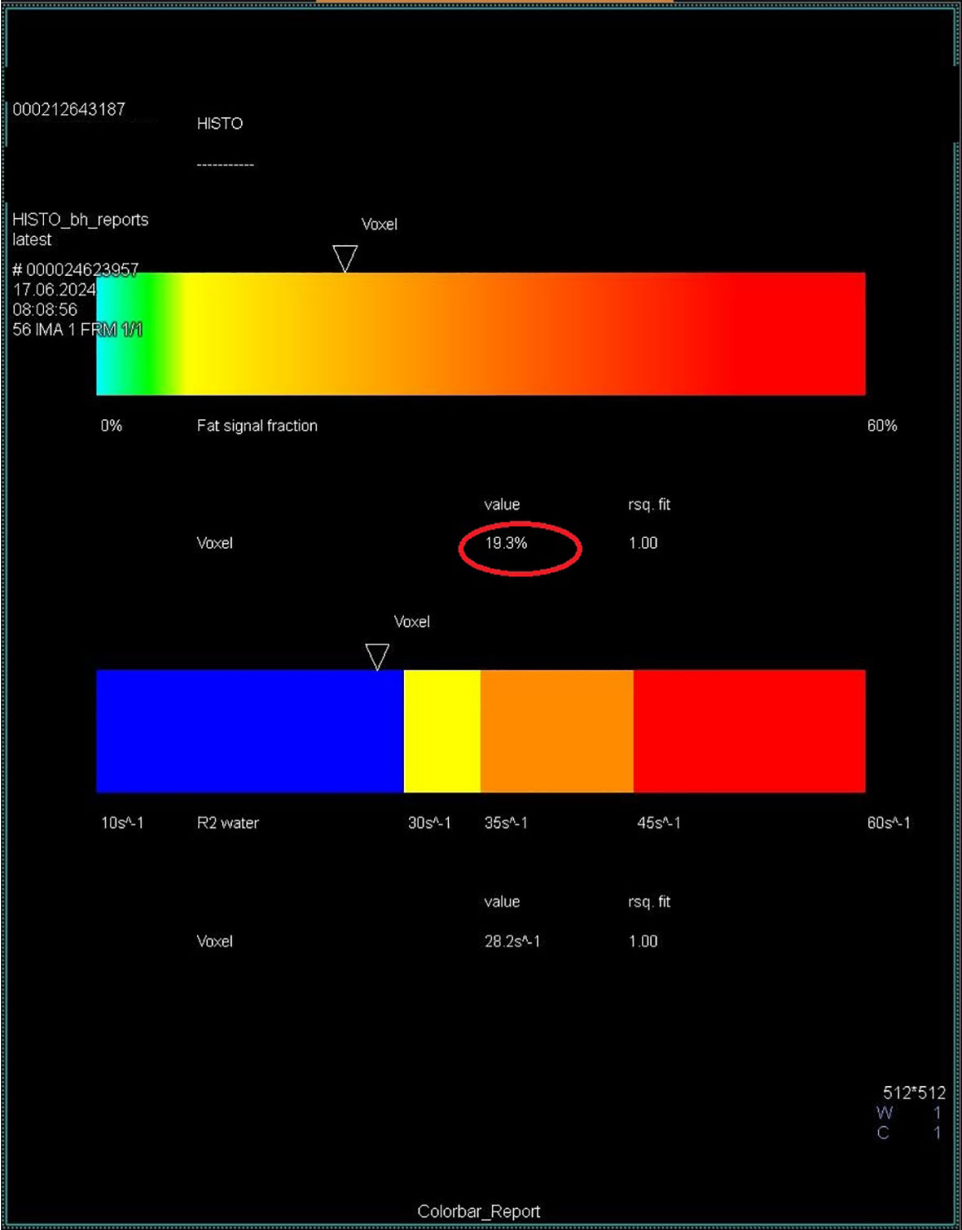
Case 2- Liver MRI image 2 (19.3% of fat content, N<5%).

**Figure 4:**
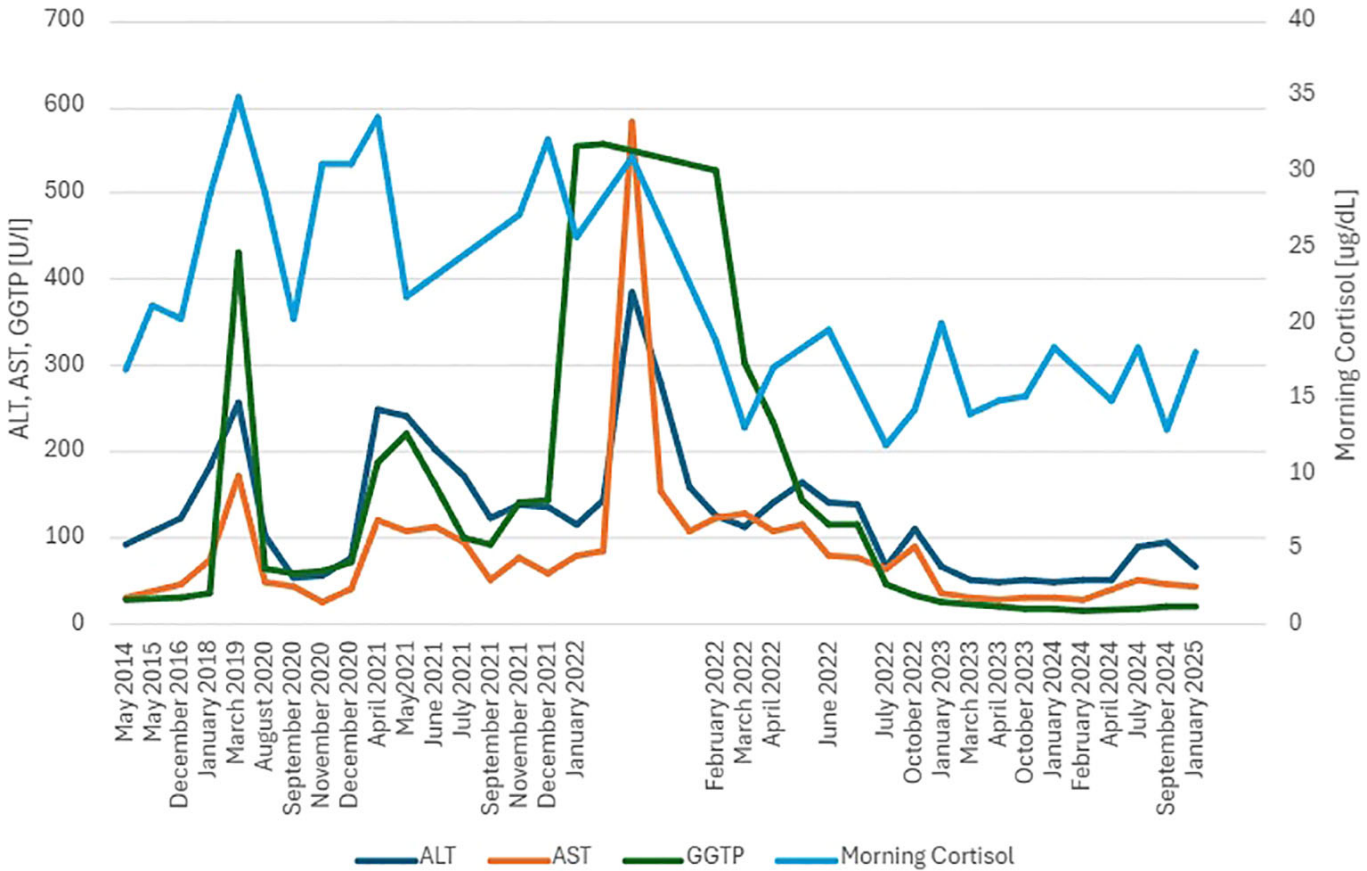
Case 2- Graph presenting LFT and cortisolemia level in the course of adrenostatic therapy.

We present a 35-year-old woman, whose case has already been published in the context of COVID-19 infection in CD treated with osilodrostat (17). In December 2021, she started osilodrostat with an initial daily dose of 2 mg followed by 4 mg since February 2022. Pre-treatment laboratory evaluation showed sustained LFT elevation (up to 4× URL), dyslipidemia, and uncontrolled DM. One month after osilodrostat implementation, the patient suffered from a COVID-19 infection, which worsened liver function. Adrenostatic treatment was interrupted during the infection period. At the time of osilodrostat resumption, LFTs were elevated to the maximum of 14.5× URL, but liver function was not decompensated (Child-Pugh A). Gamma glutamyl transferase (GGT) started to decrease after 1 month of therapy, normalizing after 9 months. Aspartate aminotransferase (AST) started to decline after 2 months of treatment, normalizing after 11 months. Alanine aminotransferase (ALT) started to decrease after 5 months of therapy. Fibrosis-4 Index [age (years) × AST (U/L)/[PLT (10^9^/L) × √ALT (U/L)]; result >1.3 predicts LF] normalized after 4 months of therapy (from 1.6 to 0.61) (18). Liver elastography 1 year after therapy initiation showed a liver stiffness value of 7.91 kPa (N < 8.27 kPa). Liver MRI presented LS at the level of 19.3% (**Figures 2** and **3**; N < 5%). The laboratory results are presented in **Table 2** and **Figure 4**.

**Table 2:** Case 2—Test results evaluating cortisolemia level, liver enzymes, diabetes mellitus, and dyslipidemia control (baseline, during adrenostatic treatment, and after surgery).

	2014	2016	2018	2019	2020	2021	12.2021 Osilodrostat start	01.2022 COVID	02.2022 Osilodrostat restart	07.2022	01.2023	04.2023	02.2024
ALT [U/L]	93	124	183	256	102	249	137	386	126	66	67	48	51
AST [U/L]	32	47	75	173	48	120	60	584	124	64	36	28	28
GGT [U/L]	28	30	37	432	64	189	145	558	528	46	27	20	16
Bilirubine [μmol/L]	5.15	3.77	3.88	4.88	5.17	7.2		7.07	4.8			4.2	4
Morning cortisol [μg/dL]	16.9	20.3	28.5	35	29	34	32.2	31	19	12	20	15	18
Midnight cortisol [μg/dL]	10.34	14.82	14.16	27	21								
mUFC [μg/day]					167			124		59			
HBA1C [%]				10.7	7.7	9.4	9.8		10.4	9.5	7		6.2
TC [mmol/L]	5.4	4.7	4.7	6.5	6.7								5.8
TAG [mmol/L]	1.27	1.15	1.46	2.36	3								1.3
Weight [kg]						150		141	142		140		145

Laboratory norms are the same as for Case 1.

## 3 Literature review

The review is presented in the form of tables (**Supplementary Files 1–4**).

### 3.1 Liver impairment in endogenous hypercortisolemia

**Supplementary File 2** presents studies evaluating liver impairment in endogenous CS and MACS.

### 3.2 HPA axis impact on liver in non-CS subjects

**Supplementary File 3** shows data on the link between HPA axis and liver impairment in normocortisolemic subjects.

### 3.3 Reviews evaluating glucocorticoids impact on liver

**Supplementary File 4** presents reviews on the glucocorticoid impact on the liver.

### 3.4 Impact of CS medical treatment on liver function

**Supplementary File 5** shows data on the impact of pharmacotherapy used in CS on the liver.

## 4 Discussion

The main subject of this case-based review is focused on the hepatic impairment in endogenous hypercortisolemia and on the impact of antihypercortisolemic pharmacotherapy on liver function. The data on that area are scarce and conflicting. For a better and more global comprehension of the studied topic, we additionally explored the understanding of HPA axis impact on liver function from different perspectives, as well as in eucortisolemic subjects. Therefore, we searched for ACTH and cortisol impact on the liver, the role of HPA axis in liver disorders, HPA axis dysregulation in liver impairment, hepatic consequences of liver glucocorticosteroid receptor (GR) stimulation and inhibition, and the role of intrahepatic cortisol concentration regulation by 11 beta-hydroxysteroid dehydrogenase type 1 (11β-HSD1) and 5β-reductase (5βR) in liver disorders.

Thus, does HPA activation promote or protect from liver impairment?

Preclinical studies showed that hepatic GR activation leads to an increase in liver fat content, most likely by the promotion of hepatic gluconeogenesis and lipogenesis (19, 20). Studies on a selective glucocorticoid receptor modulator “miricorilant” used in patients with LS/NASH revealed reduction of liver fat content (21–23). Nonclinical observations showed that miricorilant’s hepatic lipid-lowering effect resulted from a unique GR−dependent stimulation of lipid efflux from the liver, combined with a lack of stimulation of GR-dependent hepatic fatty acid uptake (21, 22). Rapid LS reduction obtained on high daily miricorilant doses resulted in the elevation of concurrent LFTs (24). The mechanism may be connected with rapid fat “wash out” from the liver, resulting in a “mechanical mini damage like process”. Subjects receiving intermittent and lower doses of miricorilant showed gradual liver fat loss without an associated rise of LFTs. Miricorilant co-administration with olanzapine showed less metabolic and hepatic side effects compared to those observed on olanzapine monotherapy (25). Reports on another GR antagonist “mifepristone” effect on the liver also seem to be inconsistent. In some cases, it improved LFTs (26), while in other cases, it was associated with liver toxicity (27). The liver safety profile of mifepristone has also been linked to the medication dose and to the rapidness of liver fat content decrease (26, 27).

Long-term intensive steroid treatment may lead to LS (20, 28–30). Data on what dose and duration of exposure of selected types of steroids may lead to the development of LS in humans are still lacking (29). Elevated liver enzymes and LS are common findings in newly diagnosed patients with CS and MACS (LS: 20%–66% and 25%–57%, respectively; an accurate prevalence of elevated LFTs in endogenous hypercortisolemia has not been reported yet) (6–13). Some of the studies report MACS and CS correlation with LS incidence (6, 7, 31), as well as positive correlation between post-DST cortisol, midnight cortisol, and hypercortisolemia duration with LS (6, 7, 13). According to one study regarding patients with MACS, cortisol level (with cutoff >2.93 μg/dL) after a 1-mg dexamethasone suppression test was an independent factor for LS diagnosed on CT (6). Based on that study, serum-based scores [Visceral Adiposity Index, Hepatic Steatosis Index, and Fatty Liver Index are based on waist circumference, body mass index (BMI), serum level of triglycerides, high-density lipoprotein cholesterol (HDL-C), ALT, AST, and GGT], which predict LS, are higher in CS, but they do not correlate with LS values on CT (6). Other studies suggest that cortisol action on LS is rather indirect via enhancing visceral fat content and worsening of glycemic and lipid control (7, 10). Mediation analysis showed that fasting plasma glucose, glycated hemoglobin (HbA1c), HDL-C, and triglyceride-glucose index (TyG) mediated the correlation between MACS or CS and the liver-to-spleen (L/S) ratio on CT (7). In this study, the only direct association between cortisolemia markers and LS was proved for midnight serum cortisolemia (7). Another study showed that L/S was correlated with abdominal and visceral fat content, but not with hypercortisolemia markers (10). Some studies reveal LS regression after CS remission and no new LS incidence after CS remission (9, 12). Other studies show no correlation between level and the duration of hypercortisolemia and LS incidence in CS (8, 12). According to some papers, there is no difference between CS types in terms of LS prevalence (12). Another study shows that LS occurs more frequently among ACTH-dependent CS, as compared to adrenal CS (11). In contrast, it has been considered that the liver impairment prevalence in hypercortisolemic patients with many metabolic comorbidities would be expected to be much higher than it has been reported (10, 14). The relatively low LS rate in CS can be explained by anti-inflammatory cortisol action, as a preventive factor from development of liver impairment in subjects with active hypercortisolemia (14). To the best of our knowledge, LF prevalence has been estimated in two studies (12, 13). Based on serum-based scores, LF occurred in 15.4% of active CS individuals (12). According to another study, in which Fibroscan was performed, LF prevalence was 3.4% in newly diagnosed CS (13). Currently, there are two ongoing prospective studies investigating liver images from MRI scans in both newly diagnosed patients with CS and those after 1 year of successful treatment (15, 16). One of these studies investigates LS by using liver MRI (15), while another measures LF using MRE (16).

Our two CS cases had LS and significant LFT elevation without hepatic function decompensation (Child-Pugh A) at baseline evaluation. Osilodrostat and metyrapone were implemented at routinely used doses. Adrenostatic therapy resulted in cortisolemia improvement, followed by a decrease in LFTs to almost normal values. These data support the hepatic safety aspects of these medications and may suggest hepatotoxic cortisol action. According to the literature, liver impairment does not require metyrapone dose adjustment (32, 33). Based on FAERS, so far there have been 41 reports of hepatobiliary adverse events associated with metyrapone (34, 35). Among 130 CS cases on metyrapone found on PubMed, 107 patients were presented without any notice regarding liver function either before, during, or after treatment with metyrapone (36–116). Nine cases have been presented only with baseline liver enzymes’ concentration, which was normal at the time of metyrapone initiation (117–125). In 11 cases, metyrapone was started regardless of liver enzyme elevation (1.5–6.4× ULN) (126–135); there was no information regarding follow-up results among these patients. Three patients presented with liver enzyme elevation during metyrapone treatment; two cases were attributed to preeclampsia (70, 136), while in another case, it was associated with rhabdomyolysis (137). Reduced osilodrostat doses are recommended only in cases with moderate or severe liver impairment (Child-Pugh B or C). Reports on LFTs after osilodrostat implementation showed either a decrease in liver enzymes or a mild and reversible increase, mainly at the beginning of therapy. LFT elevation above 5× ULN occurred when osilodrostat was used with ketoconazole. Based on FAERS, there have been seven reports of hepatobiliary adverse events associated with osilodrostat thus far (138). Ketoconazole therapy is frequently connected with hepatotoxicity (139). However, the safety profile comes mainly from the studies evaluating ketoconazole used in fungal infections (140). Ketoconazole doses used in CS are much lower. One of the studies demonstrated that liver impairment may actually improve after cortisol normalization associated with the use of ketoconazole (141). Based on FAERS, there have been 921 reports to date (in total and 35 in CS) with hepatobiliary adverse events on ketoconazole therapy. Currently, there is an ongoing KetoPASS study investigating ketoconazole liver safety profile used in CS (142). Levoketoconazole is believed to have lower hepatotoxic potential than ketoconazole. Pasireotide, cabergoline, and mitotane should not be used in severe liver impairment (Child-Pugh C). Moderate (Child-Pugh B) liver impairment requires pasireotide dose reduction. Mild-to-moderate liver impairment (Child-Pugh A and B) does not require cabergoline and mitotane dose reduction, but high patient monitoring (143–145). Etomidate dose should be reduced in cases of severe liver impairment (Child-Pugh C) (146). Among hypercortisolemia pharmacotherapy, GR antagonists (mifepristone) or modulators (miricorilant) seem to have the most divergent liver safety profile reports, which can mainly be explained by dose dependence and the rate of LS reduction.

It has been suggested that low cortisolemia may worsen outcome in patients with liver failure (147, 148). In preclinical studies, carbon tetrachloride (CCl_4_) induced irreversible liver injury in subjects with hypoadrenalism compared to subjects with normal adrenal function (149).

There are data suggesting chronic HPA axis activation in eucortisolemic obese/overweight/diabetic (DMt2) patients with LS (150–152). In these patients, post-DST cortisol and urinary free cortisol (UFC) seem to be higher than in patients without LS. Results of these studies showed that post-DST cortisol and UFC seem to predict LS in eucortisolemic individuals. In contrast, another study showed no correlation between LS and serum cortisol in healthy overweight individuals (153).

The role of intrahepatic cortisol concentration regulation in the pathogenesis of liver impairment is another interesting aspect. 11β-HSD1 increases intrahepatic cortisol concentration by cortisone-to-cortisol conversion (11b-HSD1 regenerates cortisol from circulating cortisone produced in the periphery by 11b-HSD2). 5βR deactivates cortisol and decreases its intrahepatic concentration. There are studies that show that 11β-HSD1 inhibition or 11β-HSD1 deficiency leads to hepatic glucocorticosteroid (GC) resistance and prevents LS development regardless of serum cortisol levels, and that cortisol decreases liver fat content in patients with LS (154–156), whereas 11β-HSD1 overexpression or 5βR deficiency results in intrahepatic hypercortisolemia and leads to LS (157–159). According to one study, 5βR deficiency also increases the risk of LF (157). Based on another study, 5βR deficiency protects from developing fibrotic sequelae and hepatocellular cancer in patients with LS (160). There are data that report an increased 5βR activity in patients with LS and NASH, leading to increased HPA axis activation (161), which may be in line with studies demonstrating higher cortisol levels in patients with LS/NASH (150–152). A model of LS progression to NASH based on 5βR and 11β-HSD1 activity also exists (162). According to that model, in the beginning phases of LS, there is an increased cortisol clearance (5βR overexpression) and decreased hepatic cortisol regeneration (11β-HSD1 underexpression), which is postulated to be a protective mechanism to decrease local GC availability and to preserve hepatic metabolic phenotype (162). Failure to regulate intrahepatic cortisol concentration in this way increases local GC availability and may worsen the phenotype of liver disease, leading to LS progression and inflammation. When patients develop NASH, there is an increased cortisol regeneration (11β-HSD1 overexpression) and decreased hepatic cortisol clearance (5βR underexpression), resulting in increased local GC availability, which limits further hepatic inflammation (162).

## 5 Conclusions

To conclude, there are many gaps and inconsistencies in the data regarding liver impairment (type/prevalence/pathogenesis) in endogenous hypercortisolemia and the impact of HPA axis on the liver in the general population. We can hypothesize that differences in GR sensitivity, CS and MACS heterogeneity, and many unidentified co-factors may play a role in the divergent observations. There are also some conflicting reports regarding the liver safety profile of pharmacotherapy used in hypercortisolemia. The presented CS cases showed significant baseline LFT elevation, which improved after metyrapone or osilodrostat implementation.

## Glossary

5αR: 5 alpha reductase
5βR: 5 beta reductase
6AM COR: 6 AM serum cortisol
8AM COR: 8 AM serum cortisol
11β-HSD1: 11β-hydroxysteroid dehydrogenase type 1
24AM COR: midnight cortisol
ACTH: adrenocorticotropic hormone
ALP: alkaline phosphatase
ALT: alanine aminotransferase
AST: aspartate aminotransferase
Aug: August
CD: Cushing disease
CS: Cushing syndrome
CT: computed tomography
Dec: December
DILT: drug-induced liver toxicity
DILIrank: drug-induced liver injury rank**;** FAERS, Food and Drug Agency-approved Adverse Events Reporting System
FIB4: fibrosis-4 index
GC: glucocorticoids
GR: glucocorticoid receptor
GGT: gamma glutamyl transferase
HBA1C: glycated hemoglobin
HPA: hypothalamic–pituitary–adrenal
HSI: hepatic steatosis index
HU: Hounsfield units
Jan: January
LDL: low-density lipoprotein
LE: liver enzymes
LF: liver fibrosis
LFTs: liver function tests
LNSC: late night salivary cortisol
LS: liver steatosis
mUFC: mean urinary free cortisol
M: metyrapone
MACS: mild autonomous cortisol secretion syndrome
MAFLD: metabolically associated fatty liver disease (MAFLD, MASLD, and NASLD are used interchangeably, dependent on the nomenclature changes)
MASLD: metabolically associated steatotic liver disease
MASH: metabolically associated steatohepatitis (MASH and NASH are used interchangeably, dependent on the nomenclature changes)
Mo: months
MRI: magnetic resonance imaging
MRS: magnetic resonance spectroscopy
NAFLD: non-alcoholic fatty liver disease
NASH: non-alcoholic steatohepatitis
non-HDL: non-high-density lipoprotein
Nov: November
O: osilodrostat
post-DST cortisol: morning serum cortisol concentration after 1 mg dexamethasone suppression test
TAG: triglycerides
TC: serum total cholesterol
TyG: triglyceride-glucose.
