# Supplementary material for: Liver impairment and medical management of Cushing syndrome and MACS
Source: Front Endocrinol (Lausanne). 2025 Oct 24;16:1660316. doi: 10.3389/fendo.2025.1660316 (PMC12591882; doi:10.3389/fendo.2025.1660316)
Supplement: Supplementary file 1 [file Table1.docx]

**Supplementary file 1. Child-Pugh score used to predict the prognosis of chronic liver disease.**

| Factor | 1 point | 2 ponits | 3 points |
| --- | --- | --- | --- |
| Serum total bilirubin (umol/L) | 34 | 34-51 | >51 |
| Serum albumin (g/L) | >35 | 30-35 | <30 |
| International Normalized Time (INR) | <1.7 | 1.7-2.3 | >2.3 |
| Ascites | None | Easily controlled by medications | Poorly controlled by medications |
| Hepatic encephalopathy | None | Minimal | Advanced |

| Points | Class | One-year survival | Two-year survival |
| --- | --- | --- | --- |
| 5-6 | A | 100% | 85% |
| 7-9 | B | 80% | 60% |
| 10-15 | C | 45% | 35% |
