# Supplementary material for: Liver impairment and medical management of Cushing syndrome and MACS
Source: Front Endocrinol (Lausanne). 2025 Oct 24;16:1660316. doi: 10.3389/fendo.2025.1660316 (PMC12591882; doi:10.3389/fendo.2025.1660316)
Supplement: Supplementary file 2 [file Table2.docx]

**Supplementary file 2 (re: 3.1.). Liver impairment in endogenous hypercortisolemia.**

| **Study:** | **Study type:** | **Investigated groups:** | **Investigated parameters:** | **Results:** |
| --- | --- | --- | --- | --- |
| Candemir B, Kisip K, Akın Ş, Sanal HT, Taşar M, Candemir M, Gülçelik NE. **Prevalence and Predictive Features of CT-Derived Nonalcoholic Fatty Liver Disease in Metabolically Healthy MACS.** Clin Endocrinol (Oxf). 2025 Apr;102(4):380-388. doi: 10.1111/cen.15194. Epub 2025 Jan 2. PMID: 39748255. (6) | **Cross-sectional** | **Newly diagnosed:**  **MACS** (**N: 40**, age 60.2 ± 11.1 years, 37.5% males, BMI 30.83 ± 4.7 kg/m2)  **Non-functioning adrenal adenoma** (NFAI) (N: **60**, age 58.4 ± 11.1, males 30%, BMI 29.37 ± 4.62 kg/m2) | **Non-contrast abdominal CT** (liver steatosis evaluation)**,** serum cortisol concentration after 1mg DST, non-invasive hepatic scores (HSI, FIB-4, VAI, FLI) | LS prevalence: **25% in MACS**, **5% in NFAI**  **MACS is correlated with an increased risk of LS.**  Serum **cortisol** level after  **1 mg DST** is an independent **predictor of LS** (CT) in patients with MACS (optimum cut off for post-dexamethasone cortisol >2.93 ug/dL).  MACS- higher HSI, VAI and FLI, but no correlation with liver attenuation values in CT. |
| Yu P, Yuan H, Li X, Chen H. **Impact of cortisol on liver fat and metabolic health in adrenal incidentalomas and Cushing's syndrome.** Endocrine. 2025 Jan;87(1):334-343. doi: 10.1007/s12020-024-04043-4. Epub 2024 Sep 25. PMID: 39320593. (7) | **Cross sectional** | **Newly diagnosed:**  **MACS** (**N:100**, age 54.59+/-11.80 years, males 16.0%, BMI 25.11+/- 3.32 kg/m2)  **CS** (**N:59**, males 20.3%, age 45.91 +/-13.94 years, BMI 25.33+/- 3.21 kg/m2)  **NFAI** (**N:103,** males 41.7%, age 56.98+/-12.04 years, BMI 24.94 +/- 3.26 kg/m2) | **Non-contrast abdominal CT** (liver steatosis evaluation)**,** serum metabolic and liver parameters, FIB4, morning and midnight serum cortisol, post-dexamethasone cortisol level, ACTH | LS prevalence: **66.1% in CS, 57% in MACS,** 26.2% in NFAI  **MACS and CS** were **associated with LS** adjusted to other covariates.  **Midnight cortisol** had a **direct impact** on liver fat content.  I**ndirect** impact of hypercortisolemia on liver fat content mediated by glucose and lipids metabolism. |
| Marengo M, Briet C, Munier M, Boursier J, Rodien P, Suteau V. **Fatty liver disease along Cushing's syndrome evolution.** J Clin Endocrinol Metab. 2024 Aug 28:dgae568. doi:10.1210/clinem/dgae568. Epub ahead of print. PMID: 39193719.(12) | **Cross-sectional** | **Newly diagnosed:**  **CS** patients (N:**49,** 83.7% females, age 47+/-2.5years, BMI 30+/-3kg/m2) | **Non-contrast abdominal CT** available at the moment of diagnosis (liver steatosis evaluation)**,** serum metabolic and liver parameters, non-invasive liver fibrosis scores (FIB-4, NAFLD Fibrosis Score, eLIFT) calculated in patients with LS in CT, midnight and mean serum cortisol, post-dexamethasone cortisol level, UFC. | LS prevalence (CT): **26.5%**  LF prevalence:  15.4% (based on eLIFT and NAFLD Fibrosis score; FIB4 didn’t identify LF)  After CS remission:  I. 4 LS(+) patients had follow-up imaging: 75% showed LS regression within 1-5 years after CS treatment  II. 8 LS(-) patients had follow-up imaging: no changes in liver fat content  No differences in cortisol levels and hypercortisolemia duration between patients with and without LS |
| Chen K, Chen L, Dai J, Ye H. **MAFLD in** **Patients with Cushing's Disease Is Negatively Associated with Low Free Thyroxine Levels Rather than with Cortisol or TSH Levels.** Int J Endocrinol. 2023 Apr 12;2023:6637396. doi: 10.1155/2023/6637396. PMID: 37091746; PMCID: PMC10115525. (8) | **Cross-sectional** | Active **CD**  (N:**290**, 78.28% women; age: 38.52 ± 12.84; 62.76% BMI>24 kg/m^2)^ | Assessment of liver steatosis in abdominal **ultrasonography** (USG) and thyroid hormones in active CD | **33.79%** LS prevalence in active CD  Lower FT4 levels were independently associated with higher risk of MAFLD in patients with CD.  Serum cortisol had no independent value in the diagnosis of MAFLD in patients with CD. |
| Remon P, Piñar Gutiérrez A, Venegas Moreno E,[Dios-Fuentes E](https://www.endocrine-abstracts.org/search?a=1&q=Elena%20Dios-Fuentes), [Cano Gonzales D](https://www.endocrine-abstracts.org/search?a=1&q=David%20Cano%20Gonzales), [Romero-Gomez M.](https://www.endocrine-abstracts.org/search?a=1&q=Manuel%20Romero-Gomez) **MAFLD prevalence in a cohort of patients with Cushing’s disease.** Endocrine Abstracts. 2023, doi: [10.1530/endoabs.90.ep794](http://dx.doi.org/10.1530/endoabs.90.ep794). (13) | **Cross-sectional** | Newly diagnosed **CD**  (N: **59**, 88.1% females; age 52 [43–61] years; obesity 48.3%) | **Fibroscan**- evaluation of hepatic steatosis (CAP measured in dB/m) and liver fibrosis (measured in kPa) | LS- **55.9%** of CD patients (median CAP of 265 [212–288] db/m).  Liver fibrosis-  **3.4%** of CD patients (stage F3-F4).  LS was associated with obesity, time of hypercortisolism, curation, type 2 DM and hypertrigliceridemia. |
| Hamimi A, Abdul Sater Z, McGlotten R, MD, Matta J, Pierce A, Abd-elmoniem, Ouwerkerk R, Nieman L, Gharib A. **The Improvement in** **Hepatic Steatosis After Cushing’s Syndrome Treatment Is an Early Sign of Metabolic Recovery.** Journal of the Endocrine Society, Volume 5, Issue Supplement_1, April-May 2021, Page A98, [https://doi.org/10.1210/jendso/bvab048.196](https://doi.org/10.1210/jendso/bvab048.196.) (9) | **Interventional** | Newly diagnosed **CS**  (N:**41**, age 44±1.8 years; females 85%; BMI 32.6±1.5 kg/m²) | Liver steatosis evaluation in **Magnetic Resonance Spectroscopy** (MRS) at baseline, 6 and 12months after successful treatment of CS.  Proton Density Fat Fraction (PDFF) measurement, liver steatosis diagnosed when PDFF>5%. | Baseline: **32%** patients had liver steatosis; mean PDFF 10.4±1.7; PDFF correlated positively with BMI  6 months after treatment: PDFF decrease (-52%); LS **13%**, BMI decrease (-9%)  12 after treatment: PDFF decrease (-50%); LS **11%**; BMI decrease (-12%), HBA1C decrease (-12%).  Liver fat decreases by 6 months after normalization of cortisol and precedes the improvement of HBA1C. |
| Zhou J, Zhang M, Bai X, Cui S, Pang C, Lu L, Pang H, Guo X, Wang Y, Xing B. **Demographic Characteristics, Etiology, and Comorbidities of Patients with Cushing's Syndrome: A 10-Year Retrospective Study at a Large General Hospital in China.** Int J Endocrinol. 2019 Feb 19;2019:7159696. doi: 10.1155/2019/7159696. PMID: 30915114; PMCID: PMC6399544 (11) | **Cross-sectional** | **Newly diagnosed CS** patients (N:**1652**, 78% females, age 38.0 ± 13.6 years) | Analysis of demographic characteristics, etiology, and comorbidity data. LS- based on imaging studies. | LS prevalence:  **24.8%**- overall  27.6%- ACTH dependent CS  18.2%- ACTH independent  27.4%- CD  27.6%- EAS  40%- non-localized ACTH dependent  14.5%-adrenal adenoma  30.4%- BMAH  30%- PPNAD  LS is more frequent in males. |
| Rockall AG, Sohaib SA, Evans D, Kaltsas G, Isidori AM, Monson JP, Besser GM, Grossman AB, Reznek RH. **Hepatic steatosis in Cushing's syndrome: a radiological assessment using computed tomography. Eur J Endocrinol.** 2003 Dec;149(6):543-8. doi: 10.1530/eje.0.1490543. PMID: 14640995. (10) | **Cross-sectional** | Newly diagnosed **CS**  (N:**50**, 84% females; age 46.6 [14-79] years; 78% CD, 12% adrenal CS, 8% ectopic CS, 2% unknown origin; BMI: men 25.7+/-3.3 kg/m², women 29.3+/-7.2 kg/m²) | Evaluation of liver steatosis in newly diagnosed CS patients, based on abdominal **Computed Tomography** (CT) scans available for retrospective analysis (maximal time between CT and hormonal evaluation- 4weeks).  Liver-to-spleen CT attenuation ratio (L/S) <1 indicated liver steatosis. | **20%-** LS prevalence in active CS  L/S correlated positively with alkaline phosphatase levels but with no other CS markers and other liver enzymes.  LS was correlated with abdominal and visceral fat content.  Question to be investigated: Is that visceral fat increase in CS cause of LS rather than a direct cortisol effect on liver? |
| Official title: **Prévalence De La Stéato-fibrose Hépatique Dans Le Syndrome** **De Cushing**;  Brief title: **NAFLD and Cushing (NAC)**University Hospital, Angers ClinicalTrials.gov ID NCT05881005. (15) | **Clinical trial** | **100** newly diagnosed **CS** patients | **Hepatic Magnetic Resonance Imaging** (MRI)- liver steatosis evaluation at the baseline and 1 year after CS treatment | Still recruting; estimated study completion time 09.2027 |
| Central Hospital, Nancy, France. **Evaluation of the Severity of Hepatic Fibrosis by Magnetic Resonance Elastography in the Diagnosis of Endogenous Hypercorticism (HEPACORT).** ClinicalTrials.gov ID NCT05911620. (16) | **Clinical trial** | N:**21**  3 groups: newly diagnosed **CS** patients, **possible CS** patients (plasma cortisol after 1 mg dexamethasone supression test: 1.8-5 ug/dL), **metabolic syndrome group** | **Hepatic Magnetic Resonance Elastography** (MRE)- liver fibrosis evaluation at the baseline | Still recruting; estimated study completion time 08.2026 |
