# Supplementary material for: Liver impairment and medical management of Cushing syndrome and MACS
Source: Front Endocrinol (Lausanne). 2025 Oct 24;16:1660316. doi: 10.3389/fendo.2025.1660316 (PMC12591882; doi:10.3389/fendo.2025.1660316)
Supplement: Supplementary file 3 [file Table3.docx]

**Supplementary file 3 (re: 3.2.) HPA axis impact on liver in non-CS subjects.**

| **Study:** | **Study type:** | **Investigated groups:** | **Investigated parameters:** | **Results:** |
| --- | --- | --- | --- | --- |
| **ACTH-LIVER** |  |  |  |  |
| BONGIOVANNI AM, BLONDHEIM SH, EISENMENGER WJ, KUNKEL HG. **Effects of ACTH in patients with liver disease.** J Clin Invest. 1950 Jun;29(6):798. PMID: 15436683. (151) | **Interventional** | Patients with **liver cirrhosis** (N:**7**) treated with ACTH administration. | Liver serum parameters evaluated in the course of ACTH administration (50-100mg daily for 6-21 days). | Patients with billiary cirrhosis and severe alcoholic cirrhosis with ascites didn’t show improvement.  4 females (age<35) with cirrhosis of undetermined etiology showed bilirubin drop and albumine increase which persisted 6 weeks after therapy was stopped.  AE occured in 4 of the more severely ill patients: bloody ascites (N:2), elevation of fasting glycemia >250mg% (N:2). |
| **CORTISOL-LIVER** |  |  |  |  |
| Zhang J, Li J, Ding M, Chen Y, Duan Z. **Cortisol in Peripheral Blood Predicts the Severity and Prognosis in Patients with Liver Failure at 90 Days.** Risk Manag Healthc Policy. 2021 Oct 16;14:4311-4319. doi: 10.2147/RMHP.S327440. PMID: 34703342; PMCID: PMC8528544. (135) | **Prospective observational** | Patients with **liver failure** (N: 211) | Evaluating 90 days prognosis based on serum cortisol. | The level of cortisol is correlated with the severity of liver failure and decreases with the aggravation of liver failure.  Survival group cortisol (339.26 ± 121.19 nmol/L) >non-survival group cortisol (192.71 ± 67.23 nmol/L) |
| Guarnotta V, Mineo MI, Radellini S, Pizzolanti G, Giordano C. **Dual-release hydrocortisone improves hepatic steatosis in patients with secondary adrenal insufficiency: a real-life study.** Ther Adv Endocrinol Metab. 2019 Aug 28;10:2042018819871169. doi: 10.1177/2042018819871169. PMID: 31489172; PMCID: PMC6713956. (152) | I**nterventional** | Secondary adrenal insufficiency patients (N: 45) | Impact of switching conventional HCT to dual-release HCT on liver steatosis based on HSI. | Dual-release HCT therapy resulted in improvement of LS based on HSI. |
| Hubel JM, Schmidt SA, Mason RA, Haenle MM, Oeztuerk S, Koenig W, Boehm BO, Kratzer W, Graeter T, Flechtner-Mors M; EMIL-Study Group. **Influence of plasma cortisol and other laboratory parameters on nonalcoholic Fatty liver disease.** Horm Metab Res. 2015 Jun;47(7):479-84. doi: 10.1055/s-0034-1389982. Epub 2014 Oct 8. PMID: 25295415. (141) | **Cross-sectional study** | **1 326** healthy subjects  (49.9% women; age 41.5±12.7 years; BMI: females 24.9±5.1 kg/m^2^; males 26.2±4.2 kg/m^2^) | Liver steatosis assessment in **USG** among healthy subjects. Evaluation of connection between cortisol level and presence of LS in healthy people. | 26.4% prevalence of LS in healthy subjects.  There was no association between serum cortisol concentration and presence of LS. |
| Targher G, Bertolini L, Rodella S, Zoppini G, Zenari L, Falezza G. **Associations between liver histology and cortisol secretion in subjects with nonalcoholic fatty liver disease.** Clin Endocrinol (Oxf). 2006 Mar;64(3):337-41. doi: 10.1111/j.1365-2265.2006.02466.x. PMID: 16487446. (138) | **Cross sectional study** | **50 overweight** patients **with LS** confirmed in biopsy  (age 46 ± 4 years, BMI 26·6 ± 1·6 kg/m2)  **40 overweight** subjects **without LS** (control group)  (age 46 ± 3 years, BMI 26·2 ± 1·8 kg/m2) | 24 hour urine free cortisol (UFC), cortisol on 1mg DST, liver biopsy evaluation | Patients with LS had higher UFC (149 ± 24 vs. 90 ± 16 nmol/day) and higher postdex suppression cortisol levels (32 ± 10 vs. 16 ± 7 nmol/l) than controls.  UFC and postdex suppression cortisol level correlated positively with liver inflammation and fibrosis rate.  UFC and postdex suppression cortisol level was independent predictor of liver fibrosis, but not of LS.  Results suggest that patients with LS have chronic hypothalamic-pituitary axis overactivation. |
| Gurakuqi GC, Stadlbauer V, Stepan V, Warnkross H, Trauner M. Morbus Addison als seltene Ursache für chronisch erhöhte Transaminasen [**Addison's disease as a rare cause of chronically elevated liver enzymes**]. Z Gastroenterol. 2006 Feb;44(2):179-83. German. doi: 10.1055/s-2005-858976. PMID: 16456760. (153) | Case report with brief literature overview. | Addison’s disease | LFTs elevation at baseline evaluation of patients with Addon’s disease | Addison's disease is a rare but fully reversible cause for elevated liver enzymes. |
| Targher G, Bertolini L, Zoppini G, Zenari L, Falezza G. **Relationship of non-alcoholic hepatic steatosis to cortisol secretion in diet-controlled Type 2 diabetic patients.** Diabet Med. 2005 Sep;22(9):1146-50. doi: 10.1111/j.1464-5491.2005.01583.x. PMID: 16108840. (139) | **Cross-sectional studies** | **40** patients **DMt2** **with** **LS**  (65% women, age 48 ± 1 years, BMI 25 ± 1 kg/m2**)**  **40** patients with **DMt2 without** **LS** (65% women, age 47 ± 1 years, BMI 25 ± 1 kg/m2**)** | 24 hour urine free cortisol (UFC), cortisol on 1mg DST, metabolic parameters evaluation. LS evaluation in USG. | Patients with LS had higher UFC (191 ± 4 vs. 102 ± 3 nmol/24 h; P < 0.001) and higher post-dexamethasone cortisol level (29.1 ± 2 vs. 14.4 ± 1 nmol/l; P < 0.001) than subjects without LS.  UFC and post-dexamethasone cortisol level were independent LS predictors.  LS is associated with hypothalamic-pituitary axis activation in type-2 diabetic patients. |
| Zoppini G, Targher G, Venturi C, Zamboni C, Muggeo M. **Relationship of nonalcoholic hepatic steatosis to overnight low-dose dexamethasone suppression test in obese individuals.** Clin Endocrinol (Oxf). 2004 Dec;61(6):711-5. doi: 10.1111/j.1365-2265.2004.02154.x. PMID: 15579185. (138) | **Cross sectional study** | **54** obese patients  (31.5%females, age M: 36 ± 3, F: 41 ± 2 years, BMI M: 39 ± 2, F: 41 ± 1 kg/m^2^) | Liver **ultrasonography**, serum tests, **cortisol after 1mg dexamethasone supression test** (DST) | 72%- LS  Subjects with LS had higher cortisol concentration on 1 mg DST vs without LS (21.9 +/- 2.6 vs. 11.0 +/- 1.4 nmol/l, P < 0.001).  Regression analysis showed that cortisol levels on DST was an independent LS predictor.  LS is associated with hypothalamic-pituitary axis activation in obese people. |
| **GC** **receptors-LIVER** |  |  |  |  |
| Alkhouri N, Rudraraju M, Kowdley K, Leibowitz M, Benun J, Jenders R, et al. **Miricorilant Reduced Liver Fat and Cardiometabolic Disease Markers in a Phase 1b, Open-Label Dose-Finding Study in Patients with Non-Alcoholic Steatohepatitis.** Abstract NASH-TAG 2024 Congress (21) | **Phase 1b, Open-Label Trial.** | Patients with NASH/MASH  (N:63) | Exploring if significantly lower doses and intermittent dosing of miricorilant could gradually reduce liver fat content without a corresponding rise in liver enzymes (exploring the impact on liver based on different miricorilant dosing pattern). | 100 mg TWICE WEEKLY was safe, well-tolerated, and resulted **in reduced liver fat content and improved hepatic markers.** This dosing schedule resulted in gradual reduction in liver fat of ~30% over 12 weeks without an associated rise in LFTs.  Subjects receiving **intermittent miricorilant** lost liver fat content more gradually and were less likely to have a rise in ALT >3× ULN compared to daily dosing.  Patients with drug held due to LFTs increase did not have a secondary rise in ALT when miricorilant was restarted, suggesting ALT increase was transient. |
| Alkhouri et al.  **A Phase 2a, Randomized, Double-Blind, Placebo-Controlled Study Evaluating the Safety, Efficacy, and Pharmacokinetics of Miricorilant in Patients with Presumed Nonalcoholic Steatohepatitis** (NCT03823703).  NASH-TAG 2023 Congress (23) | **A double-blind, multi-center, placebo-controlled, randomized 3-arm phase 2a.** | Patients with NASH/MASH (N:24) | To evaluate the safety and efficacy of miricorilant in decreasing liver fat content in patients with presumed NASH/MASH. 3 groups miricorilant 900mg/daily, miricorilant 600mg/daily, placebo. | 4 patients presented rapid reductions (–39% to –74%) in liver fat content (treatment time 30-44 weeks).  They experienced concurrent elevations in serum ALT and AST levels (>5× ULN), leading to early study termination by the sponsor.  LFTs increase resolved rapidly in all patients upon discontinuation of miricorilant. |
| Kroon J, Gentenaar M, Moll TJA, Hunt H, Meijer OC. **Glucocorticoid receptor modulator CORT125385 alleviates diet-induced hepatosteatosis in male and female mice.** Eur J Pharmacol. 2023 Oct 15;957:176012. doi: 10.1016/j.ejphar.2023.176012. Epub 2023 Aug 25. PMID: 37634839. (22) | **Preclinical study.** | Mice with LS. | Effect of CORT125385 (miracorilant) and mifepristone on mice LS. | Miracorilant: improved LS in female and male mice.  Mifepristone: improved LS only in female mice. |
| Hunt HJ, Donaldson K, Strem M, Tudor IC, Sweet-Smith S, Sidhu S. **Effect of Miricorilant, a Selective Glucocorticoid Receptor Modulator, on Olanzapine-Associated Weight Gain in Healthy Subjects:** A Proof-of-Concept Study. J Clin Psychopharmacol. 2021 Nov-Dec 01;41(6):632-637. doi: 10.1097/JCP.0000000000001470. PMID: 34369902; PMCID: PMC8575171. (24) | **Single-site, randomized, double-blind placebo-controlled trial.** | Healthy men (N:66) | To determine if coadministration of miricorilant with olanzapine would decrease the rate of metabolic complications of olanzapine. | In comparison to placebo group, coadministration of miricorilant with olanzapine was correlated with smaller increases in AST (difference, −32.24 IU/L; *P* = 0.009) and ALT (difference, −49.99 IU/L; *P* = 0.030). |
| Marino JS, Stechschulte LA, Stec DE, Nestor-Kalinoski A, Coleman S, Hinds TD Jr. **Glucocorticoid Receptor β Induces Hepatic Steatosis by Augmenting Inflammation and Inhibition of the Peroxisome Proliferator-activated Receptor (PPAR) α**. J Biol Chem. 2016 Dec 9;291(50):25776-25788. doi: 10.1074/jbc.M116.752311. Epub 2016 Oct 26. PMID: 27784782; PMCID: PMC5203696. (19) | **Preclinical study.** | Obese mice on high-fat diet. Mice with GRβ overexpression. | The aim of this study was to determine whether GRβ inhibits the actions of GCs in the liver, or enhances hepatic lipid accumulation. | GRβ expression increases in adipose and liver tissues in obese high-fat diet fed mice.  Overexpression of hepatic GRβ increases inflammation pathways in liver, which leads to hepatic lipid accumulation. |
| Corcept Therapeutics. **A Phase 2b, Study Evaluating Miricorilant in Adult Patients with Nonalcoholic Steatohepatitis/ Metabolic Dysfunction-Associated Steatohepatitis (MONARCH).** ClinicalTrials.gov ID NCT06108219. Updated December 10, 2024. Assessed February 24, 2025. (154) | **A Phase 2b, Randomized, Double-Blind, Placebo-Controlled** | Patients with NASH/MASH (estimated number 75) | Miricorilant 100 mg TWICE WEEKLY- impact on liver. | Still recruting |
| **11β-HSD1 and 5αR-LIVER** |  |  |  |  |
| Yadav Y, Dunagan K, Khot R, Venkatesh SK, Port J, Galderisi A, Cobelli C, Wegner C, Basu A, Carter R, Basu R. **Inhibition of 11β-Hydroxysteroid dehydrogenase-1 with AZD4017 in patients with nonalcoholic steatohepatitis or nonalcoholic fatty liver disease: A randomized, double-blind, placebo-controlled, phase II study.** Diabetes Obes Metab. 2022 May;24(5):881-890. doi: 10.1111/dom.14646. Epub 2022 Jan 25. PMID: 35014156; PMCID: PMC9135169. (142) | **A Phase 2, Randomized, Double-Blind, Placebo-Controlled** | 93 patients with LS/NASH and with or without DMt2, randomly assigned to study drug/placebo (1:1) | Liver fat content in MRI and hepatic cortisol to cortisone conversion evaluation at baseline and 12 months after treatment with AZD4017  (selective 11β-HSD1 inhibitor)/placebo | Liver fat content decreases in the group NASH+Dmt2 who received AZD4017, but not in placebo group.  Hepatic cortisol to cortisone conversion inhibition in all patients who received AZD4017.  No differences in fat content decrease between whole group treated with AZD4017 and placebo.  No impact on liver fibrosis. |
| Livingstone DE, Barat P, Di Rollo EM, Rees GA, Weldin BA, Rog-Zielinska EA, MacFarlane DP, Walker BR, Andrew R. **5α-Reductase type 1 deficiency or inhibition predisposes to insulin resistance, hepatic steatosis, and liver fibrosis in rodents**. Diabetes. 2015 Feb;64(2):447-58. doi: 10.2337/db14-0249. Epub 2014 Sep 19. PMID: 25239636. (145) | **Basic study** | **5αR1 knockout [KO] mice** | Liver examination after metabolic (high-fat diet) and fibrotic (carbon tetrachloride [CCl4]) challenge. | 5αR1 deficiency induced insulin resistance and LS, consistent with the liver accumulation of glucocorticoids, and predisposed to liver fibrosis. |
| Stefan N, Ramsauer M, Jordan P, Nowotny B, Kantartzis K, Machann J, Hwang JH, Nowotny P, Kahl S, Harreiter J, Hornemann S, Sanyal AJ, Stewart PM, Pfeiffer AF, Kautzky-Willer A, Roden M, Häring HU, Fürst-Recktenwald S. **Inhibition of 11β-HSD1 with RO5093151 for non-alcoholic fatty liver disease: a multicentre, randomised, double-blind, placebo-controlled trial.** Lancet Diabetes Endocrinol. 2014 May;2(5):406-16. doi: 10.1016/S2213-8587(13)70170-0. Epub 2014 Feb 17. PMID: 24795254. (143) | **Clinical trial (phase 1b)** | 82 patients with LS randomly assigned to study drug/placebo (1:1) | Liver fat content evaluation in MRS at baseline and 12 months after treatment with RO5093151 (11β-HSD1 inhibitor)/placebo | Liver fat content decrease in the RO5093151 group (from 16.75% to 14.28%), but not in the placebo group. |
| Dowman JK, Hopkins LJ, Reynolds GM, Armstrong MJ, Nasiri M, Nikolaou N, van Houten EL, Visser JA, Morgan SA, Lavery GG, Oprescu A, Hübscher SG, Newsome PN, Tomlinson JW. **Loss of 5α-reductase type 1 accelerates the development of hepatic steatosis but protects against hepatocellular carcinoma in male mice.** Endocrinology. 2013 Dec;154(12):4536-47. doi: 10.1210/en.2013-1592. Epub 2013 Sep 30. PMID: 24080367; PMCID: PMC4192287. (148) | **Preclinical.** | 5αR1^−/−^, 5αR2^−/−^, and wild-type (WT) mice fed with normal and American lifestyle-induced obesity syndrome (ALIOS) diet.  Evaluation of 5αR1 and 5αR1 expression in livers of humans with LS and without LS. | Investigation of 5αR1 role in the pathogenesis of LS. | 5αR1 deletion accelerates the development of LS but may protect against the development of LS related hepatocellular neoplasia. |
| Ahmed A, Rabbitt E, Brady T, Brown C, Guest P, Bujalska IJ, Doig C, Newsome PN, Hubscher S, Elias E, Adams DH, Tomlinson JW, Stewart PM. **A switch in hepatic cortisol metabolism across the spectrum of nonalcoholic fatty liver disease.** PLoS One. 2012;7(2):e29531. doi: 10.1371/journal.pone.0029531. Epub 2012 Feb 20. PMID: 22363403; PMCID: PMC3282715. (150) | **Prospective.** | **16** patients with **LS and elevated liver enzymes**  **32 obese** subjects with **normal liver enzymes** (control group) | Hepatic GC metabolism characteristic in patients with LS and NASH  Assessment of 11β-HSD1 and 5αR expression; assessment of urine and plasma cortisol generation profile following oral cortisone. | LS: increased cortisol clearance (5αR overexpression) and decreased hepatic cortisol regeneration (11β-HSD1 underexpression) -a protective mechanism to decrease local GC availability to preserve hepatic metabolic phenotype  Failure to regulate in this way may worsen the phenotype of liver disease (increase of local GC availability, LS progression, inflamation).  NASH: increased cortisol regeneration (11β-HSD1 overexpression) and decreased hepatic cortisol clearance (5αR underexpression), increased local GC availability- limiting hepatic inflammation |
| Candia R, Riquelme A, Baudrand R, Carvajal CA, Morales M, Solís N, Pizarro M, Escalona A, Carrasco G, Boza C, Pérez G, Padilla O, Cerda J, Fardella CE, Arrese M. **Overexpression of 11β-hydroxysteroid dehydrogenase type 1 in visceral adipose tissue and portal hypercortisolism in non-alcoholic fatty liver disease.** Liver Int. 2012 Mar;32(3):392-9. doi: 10.1111/j.1478-3231.2011.02685.x. Epub 2011 Dec 4. PMID: 22136330. (146) | **Preclinical study and interventional study** | Obese mice and  **49** patients with non-CS morbid **obesity** undergoing bariatric surgery  (71.4% females; age 42.2 [25–64 year] years; BMI 41.9 ± 6 kg/m2) | **11β-HSD1** expression analysis in tissues (liver, subcutaneous adipose tissue, visceral adipose tissue); liver steatosis assessment in **liver biopsy**; in mice evaluation of portal and peripheral corticosterone levels. | Mice: LS is associated with 11β-HSD1 overexpression in visceral adipose tissue and portal hypercortisolemia  People: LS is associated with 11-beta HSD-1 overexpression in visceral adipose tissue |
| Konopelska S, Kienitz T, Hughes B, Pirlich M, Bauditz J, Lochs H, Strasburger CJ, Stewart PM, Quinkler M. **Hepatic 11beta-HSD1 mRNA expression in fatty liver and nonalcoholic steatohepatitis.** Clin Endocrinol (Oxf). 2009 Apr;70(4):554-60. doi: 10.1111/j.1365-2265.2008.03358.x. PMID: 18665910. (149) | **Interventional** | **75** subjects with elevated liver enzymes  (63% females) | **Liver biopsy:** assessment of liver steatosis and hepatitis, evaluation of 11β-hydroxysteroid dehydrogenase type1 (11β-HSD1) and hexose6-phosphate-dehydrogenase (H6PDH) mRNA expression  Evaluation of **cortisol metabolites in urine** | Positive association between  expression of hepatic 11βHSD1 and H6PDH.  11β-HSD1 gene expression doesn’t seems to be involved in the pathogenesis of fatty liver or NASH.  Patients with LS/NASH:  showed an elevated 5α- and 5β-reduction of cortisol leading to an increased cortisol turnover rate and an activation of the HPA axis. |
| Paterson JM, Morton NM, Fievet C, Kenyon CJ, Holmes MC, Staels B, Seckl JR, Mullins JJ. **Metabolic syndrome without obesity: Hepatic overexpression of 11beta-hydroxysteroid dehydrogenase type 1 in transgenic mice.** Proc Natl Acad Sci U S A. 2004 May 4;101(18):7088-93. doi: 10.1073/pnas.0305524101. Epub 2004 Apr 26. PMID: 15118095; PMCID: PMC406470. (147) | **Preclinical study** | Apo-E transgenic (TG) **mice** with **hepatic 11β-HSD1 overexpression** | Blood analysis, hepatic 11β-HSD1 expression measurment, hepatic lipid content | Elevated hepatic 11β-HSD1 may be related with LS, insulin-resistance and hypertension in non-obese subjects.  Apo-E TG mice displayed: liver steatosis, insulin resistance, hypertension. They presented normal serum cortisol concentration and elevated liver cortisol level. |
| Kotelevtsev Y, Holmes MC, Burchell A, Houston PM, Schmoll D, Jamieson P, Best R, Brown R, Edwards CR, Seckl JR, Mullins JJ. **11beta-hydroxysteroid dehydrogenase type 1 knockout mice show attenuated glucocorticoid-inducible responses and resist hyperglycemia on obesity or stress.** Proc Natl Acad Sci U S A. 1997 Dec 23;94(26):14924-9. doi: 10.1073/pnas.94.26.14924. PMID: 9405715; PMCID: PMC25139. (144) | **Preclinical stud**  **y** | **11β-HSD1 deficient mice** | The role of 11β-HSD1 in the development of metabolic complications | Despite compensatory adrenal hyperplasia and increased adrenal secretion of corticosterone, 11β-HSD1 deficient mice showed attenuated activation of enzymes stimulated by GC, which suggests relative intrahepatic glucocorticoid deficiency. |
