# Supplementary material for: Liver impairment and medical management of Cushing syndrome and MACS
Source: Front Endocrinol (Lausanne). 2025 Oct 24;16:1660316. doi: 10.3389/fendo.2025.1660316 (PMC12591882; doi:10.3389/fendo.2025.1660316)
Supplement: Supplementary file 4 [file Table4.docx]

**Supplementary file 4 (re: 3.3.) Reviews evaluating glucocorticoids impact on liver.**

| **Study:** | **Number of reviewed publications:** | **Explored topic:** | **Results:** |
| --- | --- | --- | --- |
| Suwała S, Junik R. **Metabolic-associated fatty liver disease and the role of hormones in its aetiopathogenesis.** Endokrynol Pol. 2024;75(3):237-252. doi: 10.5603/ep.99689. Epub 2024 Jun 26. PMID: 38923899. (30) | 196 | Relationship between LS and endocrinopathies.  Proposal for classifying endocrine diseases into those having a possible, potentially strong and clear association with LS. | Hypercortisolemia was classified in the group of potentially strong relationship with LS. |
| Polyzos SA, Targher G. **Role of Glucocorticoids in Metabolic Dysfunction-Associated Steatotic Liver Disease.** Curr Obes Rep. 2024 Jun;13(2):242-255. doi: 10.1007/s13679-024-00556-1. Epub 2024 Mar 8. PMID: 38459229; PMCID: PMC11150302. (27) | 97 | Summarizing published data on the relationship between GC and MASLD. | GC are closely linked to MASLD pathophysiology, with specific clinical and therapeutic implication. |
| Trivedi HD, Lopes EW, Glissen Brown J, Dudani S, Lai M, Feuerstein JD, Pierce TT. **Steroid Use and Risk of Nonalcoholic Fatty Liver Disease in Patients with Inflammatory Bowel Disease: Systematic Review and Meta-analysis.** J Clin Gastroenterol. 2023 Jul 1;57(6):610-616. doi: 10.1097/MCG.0000000000001727. PMID: 35648974. (155) | 32 | LS in exogenous CS- prevalence of LS in patients with IBD using systemic GC. | GC were not associated with developing of LS. |
| Jarmakiewicz-Czaja S, Sokal A, Pardak P, Filip R. **Glucocorticosteroids and the Risk of NAFLD in Inflammatory Bowel Disease.** Can J Gastroenterol Hepatol. 2022 May 11;2022:4344905. doi: 10.1155/2022/4344905. PMID: 35600209; PMCID: PMC9117063. (28) | 150 | LS in exogenous CS. | There are many risk factors (e.g.protein and energy malnutrition) for developing LS in IBD patients.  Long-term intensive steroid treatment may lead to LS. There is still a lack of data on what dose and duration of exposure of selected types of steroids may lead to the development of LS in humans. |
| Brian J Wentworth, Helmy M Siragy, **Adrenal Insufficiency in Cirrhosis,** Journal of the Endocrine Society, Volume 6, Issue 10, October 2022. (136) |  |  |  |
| Rahimi L, Rajpal A, Ismail-Beigi F. **Glucocorticoid-Induced Fatty Liver Disease.** Diabetes Metab Syndr Obes. 2020 Apr 16;13:1133-1145. doi: 10.2147/DMSO.S247379. PMID: 32368109; PMCID: PMC7171875. (29) | 136 | LS in exogenous CS.  Understanding of the dose and duration of GK treatment, which leads to LS and the reversibility of the condition. | Exposure to excess GC leads to LS.  The degree of reversibility of GC-induced LS and the time course of such reversal upon discontinuation of GK are unknown. |
| Rucha Patel, Jasmine Williams-Dautovich, Carolyn L. Cummins, **Minireview: New Molecular Mediators of Glucocorticoid Receptor Activity in Metabolic** Tissues, *Molecular Endocrinology*, Volume 28, Issue 7, 1 July 2014, Pages 999-101. (20) | 145 | Molecular effects of GR activation in the liver, adipose tissue, muscle, and pancreas. | GR activation results in increase of liver lipogenesis and gluconeogenesis, causing liver steatosis. |
| Tarantino G, Finelli C. **Pathogenesis of hepatic steatosis: the link between hypercortisolism and non-alcoholic fatty liver disease.** World J Gastroenterol. 2013 Oct 28;19(40):6735-43. doi: 10.3748/wjg.v19.i40.6735. PMID: 24187449; PMCID: PMC3812473. (14) | 96 | Attempt to explain relatively low prevalence of liver steatosis in active CS. | Relatively low incidence of LS in CS may be explained by anti-inflammatory cortisol effect (inhibition of chronic inflammation mediated by Interleukin 6) |
| Ducci H, Katz R. **Cortisone, ACTH and antibiotics in fulminant hepatitis.** Gastroenterology. 1968 Apr;54(4):Suppl:757-9. PMID: 4173367.(156) | 6 | Comatous patients with fulminant hepatitis- effect of treatment with cortisone. | Comatous patients with fulminant hepatitis- favorable effect when treated with cortisone. |
| MYREN J. **The effect of ACTH on dehydrogenase activity following liver injury in mice. I. The effect of ACTH on the liver of controls.** Acta Pathol Microbiol Scand. 1960;48:205-10. doi: 10.1111/j.1699-0463.1960.tb04760.x. PMID: 14425645. (137) | **45** | ACTH and cortisol effect on liver diseases. | ACTH and cortisol effect on liver diseases- divergent data.  Liver injury after CCl4 injection was irreversible in subjects with reduced adrenal function. It was reversible when subjects had a normal adrenal function. |
| SBOROV VM, BLUEMLE LW Jr, NEEFE JR, GYORGY P. **The clinical usefulness of ACTH and cortisone in liver disease.** Gastroenterology. 1955 May;28(5):745-58. PMID: 14380587. (157) | **14** | ACTH and cortisol therapy in liver diseases (viral hepatitis, liver fibrosis, cirrhosis, hepatic coma). | Routine therapy of liver diseases with ACTH and cortisol is not recommended due to unpredictable results. |
