# Supplementary material for: Liver impairment and medical management of Cushing syndrome and MACS
Source: Front Endocrinol (Lausanne). 2025 Oct 24;16:1660316. doi: 10.3389/fendo.2025.1660316 (PMC12591882; doi:10.3389/fendo.2025.1660316)
Supplement: Supplementary file 5 [file Table5.docx]

**Supplementary file 5 (re: 3.4.) Impact of hypercortisolemia medical treatment on liver function.**

| **Medication group:** | **Medication:** | **The impact of the medication on the liver:** | **Food and Drug Adverse Event Reporting System (FAERS)**- number of AEs from 2002-2024 reported to FDA as “hepatobilliary disorders” (158): | **Drug Induced Liver Injury** (DILI) **rank Dataset Group** (FDA classification based on potential to cause DILI: Most-DILI-concern, Less-DILI concern, No-DILI concern, Ambiguous-DILI Concern) (159): |
| --- | --- | --- | --- | --- |
| **Steroidogenesis inhibitors** | **Osilodrostat** | Osilodrostat decreases cortisol production by11-beta-hydroxylase inhibition in the adrenal cortex.  A multicentre phase III study with a double blind, randomised withdrawal phase showed that increase in liver aminotransferases was infrequent, typically mild, and reversed spontaneously or following dose adjustment (160,161). Extension study proved osilodrostat’s long term favorable live safety profile (160). Patients with mild hepatic impairment (Child-Pugh A) doesn’t require dose adjustment (161). Reduced starting dose is recommended for patients with moderate hepatic impairment (Child-Pugh B, 1mg twice daily) and for patients with severe hepatic impairment (Child-Pugh C, 1 mg once daily in the evening) (161). Patients with impaired liver function who use osilodrostat, require more frequent monitoring of adrenal function (161). Among 76 CS cases on osilodrostat found on pubmed, 35 patients were presented without any notice regarding liver function either before, during or after treatment with osilodrostat (31, 164-177). 34 cases had mild LFTs elevation during the therapy with osilodrostat, which didn’t require modification of dosing regimen (178-180). 5 cases presented decrease of liver enzymes (180). In one case osilodrostat was started when liver enzymes rose up to 3xULN on ketoconazole treatment(181). One patient with liver metastasis showed liver enzyme elevation up to 5xULN when treated with osilodrostat and ketoconazole combination (179). | 7 (0 fatal) | No data |
|  | **Metyrapone** | Metyrapone decreases cortisol production by11-beta-hydroxylase inhibition in the adrenal cortex.  Its efficacy and safety have been shown in PROMPT study (182). Metyrapone is excreted by kidneys. Dose reduction is not required in patients with impaired liver function (183,184). Among 130 CS cases on metyrapone found on pubmed, 107 patients were presented without any notice regarding liver function either before, during or after treatment with metyrapone (31-111). 9 cases have been presented only with baseline liver enzymes’ level, which was normal at the time of metyrapone initiation (112-120). In 11 cases metyrapone was started regardless of liver enzyme elevation (1.5-6.4xULN) (121-130). There was no information regarding follow up results among these patients. Three patients presented with liver enzymes elevation during metyrapone treatment- in two cases it was assigned to preeclampsia (131-132), in one case it was associated with rhabdomyolysis (133). | 41 (8 fatal) | No-DILI-concern |
|  | **Ketoconazole** | Ketoconazole decreases cortisol production by cytochrome-P450 enzymes  inhibition.  Severe hepatic injury induced by ketoconazole is rare, mostly in patients treated with ketoconazole for fungal infection, regardless of liver enzymes elevation or in patients treated with other hepatotoxic agents (133). Based on major retrospective studies on ketoconazole use in CS, an increase of liver enzymes during ketoconazole treatment was frequent, ranging from 2.6% to 18.7% of cases (185-191). It was usually mild, asymptomatic and occurs mostly within first 6 months of treatment with the medication and it usually normalizes after dose decrease or ketoconazole withdrawal (133, 185-191). In a prospective, observational, compassionate-use registry study in Cushing’s syndrome in France, liver injury occured in 8.5% of patients not treated with ketoconazole before and in 3.3% of patients treated before with another ketoconazole formulation (192). The LFTs elevation happened within first month of therapy and it was reversible on timely ketoconazole discontibuation or dose reduction. According to the experience of another leading endocrinology center, liver function tests may get better during ketoconazole treatment (134)^.^ The study suggests that in severe, life threatening Cushing’s syndrome, elevated liver enzymes shouldn’t exclude patient from starting treatment with ketoconazole (133, 134). Currently there is an ongoing multicenter observational study KetoPASS, which investigates effectivness and safety (including liver safety profile) of ketoconazole in Patients with Cushing Syndrome based on data retrieved from ERCUSYN (193).  According to European Medicine Agency recommendations, ketoconazol treatment must not be initiated in patients with liver enzyme levels above 2 times the ULN or in patients taking other hepatotoxic medications (195). Liver enzymes should be checked in a weekly manner during first month of treatment or during each first month of a new dose of ketokonazole, then in a monthly manner for 6 months (194). In the case of increase of LFTs 3 times or more than 3 times the ULN, ketoconazol should be stopped (194). When the increase of liver enzymes is less than 3 times the ULN, ketoconazol daily dose should be decreased to at least 200 mg and more frequent follow up is required (194).  Among 108 CS cases on ketoconazole found on pubmed, 90 patients were presented without any notice regarding liver function either before, during or after treatment with ketoconazole (31, 32, 41, 44, 88, 111, 126, 195-251). 3 cases were presented only with baseline liver enzymes information (within normal limits) (252-254). 2 cases didn’t show liver impairment during ketoconazole therapy (255-256). 3 cases presented mild liver impairment which didn’t require change in ketoconazole treatment regimen (257-259). 10 cases showed liver impairment which required ketoconazole treatment withdrawal (125, 126, 128, 249, 260-264)- in three of them basal liver function was normal (260-261), in one of them (128) there was 3xULN baseline LE elevation, in 5 of them there was no information about baseline liver function (125, 126, 260-264). | Total: 921 (fatal 113)  In CS: 35 (fatal 5) | Most-Dili-concern |
|  | **Levoketoconazole** | Lewoketooconazole [2S,4R-ketoconazole] is an enantiomer extracted from the ketoconazole racemat and is approved by FDA for the treatment of endogenous CS (133, 265-268). The drug is considered to be more effective and to have better safety profile than ketoconazole.  It’s efficacy and safety in management of CS has been assesed in the SONICS (phase 3, multi-center, open-label, non-randomized, single-arm study) and in the LOGICS (double-blind, placebo-controlled, randomized-withdrawal study) studies (133, 266-268)^.^ Both investigations showed congruent results. 41% patients in SONICS and 45% patients in LOGICS presented any increase of ALT above ULN (133, 266-268). 7.4% patients in SONICS and 7.2% patients in LOGICS showed ALT increase between 3 and 5x ULN. 3% patients in SONICS and 3.6% patients in LOGICS presented ALT increase above 5xULN (133, 266-268). Abnormalities above the threshold of 3xULN always occured during titration with a median onset about 2 months after treatment start. Most cases were clinically asymptomatic. All liver tests abnormalities were reversible, without any clinical sequele. In the extended evaluation of SONICS study, no patient experienced ALT or AST >3× ULN (266).  Levoketoconazole is considered to have less hepatotoxic action in comparisson with ketoconazole. In a prospective, observational, compassionate-use registry study in Cushing’s syndrome in France, 13% of Patients treated with ketoconazole (group of patients who weren’t treated with ketoconazole before and who were assessed over a similar mean period of follow-up time as in SONICS) showed ALT increase at least 5xULN, when in SONICS it was observed in 3% of patients (133, 192). To the best of our knowledge, there is single case series report of 3 CS subjects treated with lewoketoconazole (269). Based on this report, LFTs didn’t show any abnormalities during lewoketoconazole therapy (269). | 10 (0 fatal) |  |
|  | **Mitotane** | **Mitotane (o,p'-DDD)** is a steroidogenesis inhibitor (by blocking 11betahydroxylase) and cytostatic antineoplastic medication used in the treatment of Cushing syndrome and adrenocortical carcinoma (265, 270). Mitotane is metabolised through the liver (270). Cases of liver impairment (hepatocellular, cholestatic and mixed), autoimmune hepatitis (7%) were described as a possible side effect (270). Especially isloated GGT level, has been very commonly reported in mitotane- managed patients. LFTs should be regularly monitored, mainly during the first months of treatment or when dose escalation is planned. According to the “product characteristics”, the use of mitotane in patients with severe hepatic impairment is not recommended (270). Patients with mild to moderate hepatic impairment, should be taken with a special caution regarding thigh monitoring of liver function and thigh monitoring of plasma mitotane concentration (270).  Among 277 CS cases on mitotane found on PubMed database, 123 patients were presented without any notice regarding liver function either before, during or after treatment with mitotane (74, 95, 100, 245, 271-293).  1 case was presented only with baseline liver enzymes information (within normal limits) (294).  66 patients showed GGTP elevation on mitotane treatment (36% patients: 3-5xULN, 18% patients: >5x ULN, 46% no information regarding GGTP level) (295-297). Among them 15 patients had GGTP elevation at baseline evaluation with increasing levels on mitotane treatment (296).  15 patients were presented with information about aminotransferase (without aminotransferase type specification) increase on the treatment with mitotane (20% patients >3xULN, 80% no information regarding aminotrasferase level) (295, 297-300).  7 patients showed AST elevation on mitotane treatment. Among them 4 patients had AST elevation at baseline evaluation with stable levels on mitotane treatment (296).  22 patients showed ALT elevation on mitotane treatment. Among them 10 patients had ALT elevation at baseline evaluation with stable levels on mitotane treatment (69, 296, 298, 301).  7 patients showed ALP elevation on mitotane treatment. Among them 5 patients had ALP elevation at baseline evaluation with stable levels on mitotane treatment (291, 296). 2 patients had an isolated ALP rise (291).  1 patient showed bilirubine elevation on mitotane treatment. Baseline bilirubine evaluation was comparable to the levels on the treatment (296).  3 patients required mitotane withdrawal due liver enzymes impairment (1- ALT 23xULN, 1- ALT 5xULN, 1- severe liver function impairment without information about the levels of liver enzymes) (69, 296, 301).  In one patient treated with ketoconazol, metyrapone and mitotane, ketoconazole was stopped due to liver enzyme elevation. Patient stayed on metyrapone and mitotane. There was no information regarding liver enzymes’ level (126). | 80 (11 fatal) | No data |
|  | **Etomidate** | Etomidate is an imidazole derivate used intravenosuly as an anesthetic induction agent. By 11β-hydroxylase inhibition, it blocks cortisol production. It’s action is very rapid. Though etomidat is not approved by FDA in the treatment of CS, it is used off label in patients with severe CS when fast drop of cortisol is needed (265, 302). Etomidate is metabolized by liver but very rarely casues severe hepatotoxicity ^[9]^. Patients with cirrhosis require drug dose reduction (302). We found 7 published CS cases treated with etomidate. 4 patients were presented without any notice regarding liver function either before, during or after treatment with etomidate (303-306). 1 case was presented only with baseline liver enzymes information (within normal limits) (308). 2 patients had elevated liver enzymes at the moment of etomidate initiation (178, 264). In one of them ALT elevation was up to 2xULN and it normalized within treatment with etomidate (178). In the second case the level of liver impairment was not precisely described, but we know it resulted in ketoconazole withdrawal and etomidate initiation (264). | Total: 30 (5 fatal), no cases regarding CS | No data |
| **Pituitary directed medications** | **Pasierotide** | Pasireotide is a somatostatin analogue used in the pharmacoterapy of CD. It has a greater affinity for somatostatine receptor type 1,3 and 5 (SSTR1 30x, SSTR3 5x, SSTR5 40x) than the classic somatostatin analogue, octreotide.  Pasireotide is eliminated mostly by biliary excretion, (about 48%) with minor renal clearance (about 7.63%) (308, 309). Pasireotide decreases cholecystokinin secretion, inhibits gall bladder contractility and reduces bile secretion (265). Long term treatment is associated with an increased rate of cholesterol gallstones creation. Most Patients develop asymptomatic cholelithiasis (308, 309).  Clinical trials:  1. A phase I, open label, multicenter study showed that severity and incidence of adverse events were similar among groups of patients with normal liver function and mild/moderate/severe liver dysfunction (310). Pasireotide exposure in patients with mild hepatic impairement was comparable to subjects with normal liver function, when patients with moderate and severe hepatic impairment showed higher exposure to pasireotide (311).  2. The main clinical trial on pasireotide treatment, a phase III, randomized, double-blind, multicenter clinical trial, showed that mild (<3), transient and mostly asymtomatic LFTs elevations has been observed in 29% patients treated with pasireotide (311). It was usually reversible with continued treatment (311). Any patient hadn’t AST or ALT elevation over 3xULN times with a concomitant elevation of the bilirubin level (311). Of the 137 patients with a normal gallbladder on ultrasonographic examination at baseline, 6.6% and 19.7% of patients developed respectively a detectable sludge and gallstones; 4% of patients underwent cholecystectomy (311).  3. In an extension study of the main phase III trial, a gallbladder/biliary-related AEs was reported in 62.5% and liver-safety related AEs in 18.8% of patients (312).  4. A phase III, randomized, double-blind, multicenter clinical trial investigating pasireotide LAR showed gallbladder or biliary-related AEs in 34.7% of patients and liver safety-related AEs in 20% of patients (312).    According to the product characteristics (308):  1. Monitoring of liver function is recommended prior to treatment initiation, after the first two to three weeks, then monthly for three months on treatment.  2. Ultrasonographic evaluation of gallbladder is advised before pasireotyde initiation, six and twelve months after treatment begining (312). Symptomatic cases should be treated according to clinical standards. Even after cholecystectomy, stones might be formed in the common bile duct or intrahepatic ducts and cause symptoms. Product characteristic doesn’t indicate cholelithiasis as a contraindication for the treatment.  3. In patients with mild hepatic impairment (Child-Pugh A) there is no need of pasireotide dose reduction. In patients with moderate hepatic impairment (Child-Pugh B), pasireotyde dosage should be reduced. In patients with severe hepatic impairment (Child-Pugh C) pasireotyde should’t be used.  4.Later on liver enzymes should be monitored as clinically indicated.  5. People with increased values of LFTs need to be monitored strictly until results’ return to baseline levels.  6. Discontinuation of treatment is recommended when: -patient develops symptoms indicating liver dysfunction (e.g. jaundice), -if there is a sustained elevation in AST or ALT of 5 x ULN or greater, -or if ALT or AST increase greater than 3 x ULN co-occur with bilirubin elevations greater than 2 x ULN. After treatment discontinuation patients need to be followed-up until resolution. Therapy should not be restarted if liver function impairment is suspected to be linked to pasireotide.  Among 45 CS cases on pasireotide found on pubmed, 34 patients were presented without any notice regarding liver function either before, during or after treatment with pasireotide (246, 313-328). 1 case was presented only with baseline liver enzymes information (within normal limits) (329). 7 cases didn’t develop liver enzyme or gall bladder abnormalities on pasireotide treatment (112, 330-333).1 case showed increase of liver enzymes level up to 3xULN (ALT, AST) (334). Liver enzymes normalized after pasireotide dose reduction from 900ug BID to 600 ug BID. In 2 cases patients developed choledocholithiasis at the 6 month of pasireotide treatment (328, 335)^-^ in one case liver enzymes didn’t increase (328), in the second case cholestatic enzymes GGTP and ALP raised up to 17.5xULN (335). After pasireotide withdrawal and cholecystectomy, liver function normalized. | Total: 94 (fatal: 12)  In CS: 12 (fatal 0) | No data |
|  | **Cabergoline** | Cabergoline is dopamine receptor type 2 agonist, used not only in patients with prolactinoma, but also in patients with CD.  Cabergoline is metabolized by the liver, so special caution should be taken when cabergoline is used in patients with hepatic impairment (336-337). Mild to moderate liver dysfunction (Class A and B- Child Pugh score) doesn’t theoretically change cabergoline pharmacokinetics (336-337). Cabergoline should’t be used in patients with severe liver dysfunction (Class C- Child Pugh score) (336-337).  Among 99 CS cases on cabergoline found on pubmed, 96 patients were presented without any notice regarding liver function either before, during or after treatment (31,213, 218, 256, 338-362). 3 patients had normal baseline LFT, with no information regarding follow up tests (363). | Total: 106 (3 fatal)  In CS: 3 (0 fatal) | No data |
| **Glucocorticoid** | **Mifepristone** | Mifepristone blocks progesteron and glucocorticosteroids receptors.  SEISMIC trial showed LFTs improvement in CS patients treated with mifepristone (300-1200mg/daily) (364). Another trial presented    One of the preclinical studies showed liver injury improvement in obese mice treated with mifepristone (daily dose 30mg/kg) (365). Another one showed reversible, hepatotoxicity during long term therapy with high dose of mifepristone (200 mg/kg/daily) (366). The hepatotoxic mechanism is suspected to be linked with an estrogen- or androgen-like effect (367).    Among 36 CS cases on mifepristone found on pubmed, 29 patients were presented without any notice regarding liver function either before, during or after treatment (36, 100, 245, 368-377). 1 patient was presented only with baseline LFT (up to 2xULN) without any information regarding follow up tests (378). 3 patients showed DILT (females, baseline LFT: normal, LFT rose up to 8.5xULN around 90 days after mifepristone start, resolved 40 days after drug withdrawal, biopsy: 2xcholestatic liver injury and 1xcholestatic liver injury with necroinflammation) (379-381). 3 patients normalized LFT (baseline LFT 3.75-20xULN) within 1-6 months of mifepristone treatment (128, 379, 380). 2 patients showed LS improvement (379, 380). 1 had normal baseline LFT with decreasing tendency after mifepristone start (379). | Total: 77 cases (5 fatal outcome)  In CS: 49 (4 fatal) | No-Dili-concern |
| **receptor antagonists** | **Relacorilant** | Relacorilant is a selective cortisol receptor inhibitor.  Open-label phase 1 and 2 studies of relacorilant showed LFTs improvement in healthy and hepatically impaired adults and in adult patients with CS treated with relacorilant (381). |  |  |
